# Association between Matrix Metallopeptidase 9 Polymorphism and Breast Cancer Risk

**DOI:** 10.1055/s-0038-1673366

**Published:** 2018-10

**Authors:** Rogério Tadeu Felizi, Melissa Gonzalez Veiga, Ivo Carelli Filho, Ricardo Peres do Souto, César Eduardo Fernandes, Emerson Oliveira

**Affiliations:** 1 Department of Gynecology, Faculty of Medicine of ABC, Santo André, SP, Brazil; 2 Department of Biochemistry, Faculty of Medicine of ABC, Santo André, SP, Brazil

**Keywords:** MMP-9 metalloproteinase, breast cancer, genetic polymorphism, genetics, single-nucleotide polymorphisms, metaloproteinase MMP-9, câncer de mama, polimorfismo genético, genética, polimorfismos de nucleotídeo único

## Abstract

**Objective** Epidemiological studies have shown evidence of the effect of genetic variations in the pathogenesis of breast cancer and have suggested a relationship of the disease with genetic polymorphisms. Matrix metallopeptidase 9 (MMP-9) is a collagenase responsible for the degradation of type IV collagen, the major component of the basement membrane, and other essential extra cellular matrix components, being involved in the tumor cell invasion and metastasis. Our objective was to evaluate the relationship between the *MMP-9*-1562 C/T polymorphism (rs 3918242) and the risk of developing breast cancer.

**Methods** In this case-control study, the frequency of the *MMP-9*-1562 C/T polymorphism (rs 3918242) was determined in 148 women with breast cancer and 245 women without the disease. The DNA was extracted from plasma samples, and the gene was amplified by polymerase chain reaction (PCR); the presence of the polymorphism was determined using restriction enzymes.

**Results** After adjusting for confounding variables, we found that the polymorphism was not associated with the occurrence of breast cancer (odds ratio [OR] = 1.159, 95% confidence interval [CI]: 0.6625–1.997, *p* = 0.5964). We also found no association with more advanced disease, the presence of hormone receptors, human epidermal growth factor receptor 2 (HER2) overexpression, or rate of tumor cell proliferation.

**Conclusion** We did not observe a relationship between *MMP-9*–1562 C/T polymorphism (rs 3918242) and the occurrence of breast cancer.

## Abstract (pt)

**Resumo:**

**Objetivo** Estudos epidemiológicos vêm demonstrando evidências da influência de variações genéticas na patogênese do câncer de mama, e têm sugerido associação de polimorfismos com uma maior susceptibilidade à doença. A metalopeptidase de matriz 9 (MMP-9) é uma colagenase responsável pela degradação do colágeno tipo IV, o maior componente da membrana basal, e outros componentes essenciais da matriz extra celular, estando envolvido na invasão da célula tumoral e metástase. Nosso objetivo foi avaliar a associação entre o polimorfismo da 1562 C/T (rs 3918242) do gene *MMP-9* e o desenvolvimento da neoplasia mamária.

**Métodos** Neste estudo caso–controle, a frequência de polimorfismo 1562 C/T (rs 3918242) do *MMP-9* foi determinada em 148 mulheres com câncer de mama e 245 mulheres sem a doença. O DNA foi extraído do plasma e o gene foi amplificado por meio de reação em cadeia da polimerase (RCP). O polimorfismo foi determinado por enzimas de restrição.

**Resultados** O polimorfismo, após o ajuste para variáveis confundidoras, não foi associado com a ocorrência de câncer de mama (razão de possibilidade [RP] = 1,159, intervalo de confiança de 95% [IC95]: 0,6625–1,997, *p* = 0,5964). Também não foi demonstrado associação com estadiamentos mais avançados, presença de receptores hormonais, superexpressão de HER2 e tampouco com a taxa de proliferação celular do tumor.

**Conclusão** Não observamos relação entre o polimorfismo 1562 C/T (rs 3918242) do gene *MMP-9* e a ocorrência de câncer de mama.

## Introduction

Breast cancer is the most common type of cancer in the female population when disregarding cases of non-melanoma skin cancer. The mortality rate due to the disease presents an upward curve,^1^ contributing to make breast cancer a major public health problem and an important cause of mortality in adults.^2^ In 2018, 59,700 new cases were estimated in Brazil, representing an incidence rate of more than 56 cases per 100,000 women.^1^ Approximately 10 to 15% of breast cancer patients have a previous family history of the disease; however, only 5% can be explained by mutation of genes such as *BRCA1* and *BRCA2*.^3^ Regarding family risk for the development of the disease, it is necessary to consider the influence of environmental factors and genetic variations that may alter the predisposition to the risk of breast cancer.^4^

Metalloproteinases (MMPs) are a family of endopeptidases participating in the degradation of extra cellular matrix and basement membrane barriers.^5^ ^6^ ^7^ Some members of the MMPs family have been shown to promote tumor growth, angiogenesis, invasion and metastasis in cancer patients.^8^ ^9^ ^10^ Matrix metallopeptidase 9 (MMP-9) is a collagenase responsible for the degradation of type IV collagen, the major component of the basement membrane, and others essential extra cellular matrix component̀s, being involved in the tumor cell invasion and metastasis.^11^ ^12^

The gene *MMP-9* is located on chromosome region 20q11.2-q13.1.^13^ Genetic variation may alter *MMP-9* expression, influencing breast cancer susceptibility.^14^ Differential expression of *MMP-9* reflects the degree of differentiation of breast cancer cells and that its overexpression is closely related with the most aggressive subtypes.^15^

Several single-nucleotide polymorphisms (SNPs) have been reported to be correlated with tumor progression and worse disease prognosis. Among *MMP-9* polymorphisms, 1562 C/T (rs 3918242), a C to T substitution at −1562 bp, is the most studied.^14^ Researches have been performed to confirm the relationship between the pointed polymorphism and breast cancer susceptibility, however results are inconclusive.^14^

In this clinical, cross-sectional case-control study, we evaluated the potential relationship of the *MMP-9* gene polymorphism with breast cancer and aggressiveness of the disease.

## Methods

We studied 393 women, recruited between 2013 and 2015, who were followed up in the mastology sector of the division of gynecology of the Faculdade de Medicina do ABC (FMABC). The participants were divided into two groups: 148 with histologically confirmed diagnosis of breast cancer (case group) and 245 without the disease, with normal clinical and mammographic examinations (control group). For the patients with breast cancer, staging of the disease was retrieved from the patients' records, based on the tumor, node and metastasis (TNM) classification of malignant tumors. Immunohistochemical analysis of the tumor was performed to determine the presence of estrogen receptors (detected using the EP1 clone), human epidermal growth factor receptor 2 (HER2) overexpression and Ki67. Tumors exhibiting at least 1% of tumor cells staining for estrogen receptor were considered positive. Breast cancers were tested for HER2 overexpression by immunohistochemistry (IHC), and cases showing equivocal (2 + ) staining by IHC required reflex testing by HHER2 D-FISH. The Ki67 cut-off was considered 25%. Clinical data were collected with the use of a questionnaire; the following data were recorded: age, age at menarche and last menstruation, number of pregnancies, previous use of hormonal medications, breastfeeding, history of smoking, alcohol consumption and endocrine diseases. The patients included were informed about the study and signed a consent form.

Venous blood samples were collected from women of both groups, and genomic DNA was extracted using the Illustra blood genomic prep mini spin reagent kit (GE Healthcare, Chicago, IL, USA), following the manufacturer's instructions. The presence of the *MMP-9* polymorphism was determined following the PCR-RFLP procedure described by Zhang et al (1999).^16^ For the amplification of the promoter region of the gene by polymerase chain reaction (PCR), the following primers were used: 5′-GCC TGG CAC ATA GTA GGC CC-3′ e 5′- CTT CCT AGC CAG CCG GCA TC -3′. The 435-bp PCR products were treated with the Sphl restriction enzyme, and the restriction fragments were separated by electrophoresis in 2.5% agarose stained with ethidium bromide. At the end of the analysis, C/C homozygotes should present a single 435-bp band, T/T homozygotes should present two bands of 247 and 188 bp, and T/C heterozygotes should present three bands of 435, 247 and 188 bp.

To assess the association between the study groups and the categorical variables, we used the frequency chi-squared test, whereas the continuous variables were analyzed using the unpaired *t*-test. The Hardy-Weinberg equilibrium was also tested using the chi-squared test. After the stratification of the groups, the effect of the *MMP-9* polymorphism on breast cancer development was estimated by the odds ratio (OR), obtained by the binary logistic regression model, using the SPSS software, version 23.0 (IBM Corp., Armonk, NY, USA). The confidence interval adopted was 95% (95%CI), and the value for rejection of the null hypothesis was set at 0.05 or 5% (α ≤ 0.05).

The project was approved by the Research Ethics Committee of the institution under number 169/2010.

## Results

A total of 148 women were included in the case group, and 245 women were included in the control group. The clinical and epidemiological characteristics of both groups are described in Table 1. Both groups presented homogeneity for almost all of the characteristics evaluated, with similar proportions of women over 50 years of age, menopausal women and/or women who used hormone therapy in the case and control groups. The variable parity and the age at first pregnancy also showed no significant differences between the groups. Cases were more likely to use oral contraceptives than controls, with frequency of use at 22.3% and 6.1%, respectively (*p* < 0.0001). The family history of breast cancer (*p* = 0.04) was more frequent in women who presented with the disease, with a difference of almost 10% between the groups.

**Table 1:** Matrix metallopeptidase 9 polymorphism—clinical characteristics of cases and controls

Characteristics	Cases (*n* = 148)	Controls (*n* = 245)	*p*-value
Age (years)^#^	57.8 ± 0.9	59.5 ± 0.6	0.134
Age at menarche (years)^#^	12.9 ± 0.1	13.2 ± 0.1	0.059
Postmenopause*	121 (81.7%)	82. 7%	0.785
Parity*	2.6 ± 0.12	2.9 ± 0.09	0.067
Breastfeeding*	116 (78.4%)	207 (84.5%)	0.13
Age at first pregnancy^#^	23.1 ± 0.45	22.7 ± 0.3	0.54
Use of oral contraceptive*	33 (22.3%)	15 (6.1%)	< 0.0001**
Use of hormone therapy*	13 (8.7%)	36 (14.7%)	0.114
Family history of breast cancer*	25 (16.9%)	18 (7.3%)	0.004**

Continuous variables: values expressed as the mean and standard deviation; Categorical variables: values expressed as numbers and percentages; ^#^ Unpaired *t*-test; * Chi-squared test; ** Significant values

Genotyping and frequency of the alleles are described in Table 2. The distribution of the genotypes is in genetic equilibrium according to the Hardy-Weinberg principle in the case and control groups.

**Table 2:** Matrix metallopeptidase 9 polymorphism and the occurrence of breast cancer

	CC	TC + TT	OR crude (CI)	OR adjusted (CI)*
Cases	115 (82.7%)	24 (17.3%)	1.159 (0.6625–1.997) *p* = 0.5964	
Controls	186 (80.5%)	45 (19.5%)		

Abbreviations: CI, confidence interval; OR, odds ratio.

Due to the low incidence of the TT genotype in the studied population, we chose to analyze the results comparing the wild homozygous group (CC) with the polymorphic group (TC + TT). After adjusting for oral contraceptive use and family history of breast cancer, the presence of the T allele and the TT (TC + TT) genotype of the *MMP-9* polymorphism was not directly associated with tumor occurrence (OR = 1.159, 95%CI: 0.6625–1.997, *p* = 0.5964). In addition, no statistically significant difference was found between the polymorphisms when analyzing according to the staging of the disease, estrogen receptor status, HER2 overexpression or non-overexpression, or cell proliferation rate represented by Ki67, as shown in Table 3.

**Table 3:** Evaluation of matrix metallopeptidase 9 polymorphism and staging, status of estrogen receptor, HER2 and Ki67

	CC	TC + TT	*p*-value*
Staging IA–IIB Staging IIB–IV	90 (81%) 25 (89%)	21 (19%) 3 (11%)	0.4075
Estrogen receptor+	86 (81.9%)	19 (18.1%)	0.796
Estrogen receptor -	29 (85.3%)	5 (14.7%)	
HER2 +	21 (87.5%)	3 (12.5%)	0.766
HER2 -	94 (81.7%)	21 (18.3%)	
Ki67 ≤ 25%	58 (85.3%)	10 (14.7%)	0.244
Ki67 > 25%	41 (75.9%)	13 (24.1%)	

Abbreviation: HER2, human epidermal growth factor receptor 2.

* Chi-squared test

## Discussion

Hereditary predisposition to breast cancer significantly influences the screening and follow-up of women at high risk for developing the disease, enabling early diagnosis, personalized conduct and family counseling. However, in patients with a personal or family history of breast cancer, a specific genetic predisposition is identified in less than 30% of cases.^17^ Thus, it seems that the effect of low-penetrance gene polymorphisms on the risk for breast cancer is relevant only in polygenic forms.^17^

Studies have demonstrated that degradation of extra cellular matrix and basement membrane by MMPs play an important role in tumorigenesis by modulating cell proliferation, apoptosis, tumor invasion and metastasis.^18^ The role played by MMP-9 in the malignancy and the growth of the tumor is an important one.^14^

Numerous single-nucleotide polymorphisms (SNPs) have already been identified in the *MMP-9* gene, and the most common variant is 1562 C/T (rs 3918242). Our study demonstrated that the T allele and the TT genotype of the *MMP-9* polymorphism were not directly associated with the occurrence of breast cancer, as shown in Table 2.

The association between this polymorphism and the disease has already been studied by groups from several countries, without a direct relationship being established. An Indian study revealed that the frequency of T-allele of MMP-9–1562 C/T promoter polymorphism was found to be predominant in breast cancer group compared with controls, with a 1.44 folds increased risk for breast cancer.^19^ Merdad et al^20^ showed that *MMP-9* is a reliable potential candidate diagnostic biomarker and drug target in breast cancer. An Iranian study,^18^ published in 2015, found association between higher risk of developing breast cancer and *MMP-9* polymorphism, as the presence of mutant allele increased the susceptibility to the disease by 1.87 times (OR = 1.87, IC95: 1.05–3.33, *p* = 0.035). Similarly, a large meta-analysis,^7^ also from 2015, showed an association between TT genotype of *MMP-9*–1562 C/T polymorphism and a higher risk for breast cancer. This result was in contrast to our study, which was supported by a South Brazilian research (*p* = 0.23)^21^ and a meta-analysis conducted by Zhou et al,^22^ which involved more than 5,000 patients. Both studies found no evidence that the 1562 C/T polymorphism (rs3918242) is correlated with a higher risk for breast cancer.

Several studies have described the role of MMPs in breast cancer progression and metastasis development, as MMPs are involved in the degradation of basement membranes.^23^ In a meta-analysis^24^ including more than 10,000 cancer cases, *MMP-9* *(1562 C/T)* was shown to increase the risk of cancer metastasis, correlating the polymorphism with a more aggressive disease (OR = 1.25, IC95: 1.03 - 1.51, *p* = 0.07). Our results were inconsistent with this finding, as the adjustment for disease stage did not involve a higher frequency of polymorphism in more advanced tumors (*p* = 0.4075).

A factor with strong involvement that has not yet been established as a risk factor is the use of oral contraceptive, which, in our study, was related to higher occurrence of the disease (*p* < 0.0001).^25^ A meta-analysis^26^ correlating Iranian studies demonstrated that the use of oral contraceptives may stimulate the occurrence of breast cancer because it directly increases estrogen levels and indirectly influences weight gain. Our finding is consistent with the results reported in an analysis^27^ published in 2016, which showed an OR of breast cancer development that was 54.6% lower in patients who did not use oral contraceptives compared with those who used it.

Approximately 5 to 10% of breast cancer cases are familial and occur earlier than those in the general population. The BRCA1 and BRCA2 mutations are primarily responsible for hereditary breast cancer.^28^ Despite years of research, it has been shown that a minority of patients with a personal or family history of breast cancer have a genetic mutation as an identifiable cause.^17^ Our study is consistent with the global literature, as we found a positive association of family history with development of the disease (*p* = 0.004).

Stratified analysis according to HER2 or estrogen receptor expression in neoplastic cells showed no relationship with the occurrence of the polymorphism studied, and similarly, Ki67—a tumor cell proliferation index—was not a factor associated with the greater presence of the polymorphic alleles. We believe, however, that more studies are needed to confirm any of the proposed hypotheses due to the lack of evidence in the literature on the subject.

## Conclusion

The main results of this study suggest that the T allele and the TT genotype of 1562 C/T (rs 3918242) of the *MMP-9* do not play a key role in breast cancer development. Although research has been performed to explore the role of SNPs of this gene in breast cancer, the results are inconclusive. We note that the controversy remains over the influence of 1562 C/T (rs 3918242) of the *MMP-9* on the genesis of breast cancer. More studies and a larger case sample are necessary to confirm the effects on the risk of breast cancer to assist in the screening and follow-up of patients at increased risk of the disease.
